# Feed Restriction Induced Changes in Behavior, Corticosterone, and Microbial Programming in Slow- and Fast-Growing Chicken Breeds

**DOI:** 10.3390/ani11010141

**Published:** 2021-01-11

**Authors:** Chao Yan, Jinlong Xiao, Di Chen, Simon P. Turner, Zhiwei Li, Hao Liu, Wen Liu, Jian Liu, Siyu Chen, Xingbo Zhao

**Affiliations:** 1 College of Animal Science and Technology, China Agricultural University, Beijing 100193, China; ychplus@126.com (C.Y.); grdwy123456@163.com (J.X.); chendiabm@163.com (D.C.); 17600465226@163.com (Z.L.); liuhao299101@163.com (H.L.); liuwenwindy@cau.edu.cn (W.L.); 2 Guizhou Nayong Professor Workstation, China Agricultural University, Bijie 553300, China; liujian8230@163.com; 3 Animal and Veterinary Sciences Department, Scotland’s Rural College, Edinburgh EH25 9RG, UK; simon.turner@sruc.ac.uk; 4 Guangdong Provincial Key Laboratory of Animal Molecular Design and Precise Breeding, Key Laboratory of Animal Molecular Design and Precise Breeding of Guangdong Higher Education Institutes, School of Life Science and Engineering, Foshan University, Foshan 528225, China

**Keywords:** feed restriction, gut microbiota, slow- and fast-growing breeds, chronic stress, chicken

## Abstract

**Simple Summary:**

Different genotypes of slow- and fast-growing chickens have phenotypic changes in appearance, behavior, and productivity in response to artificial selection. Feed restriction and gut microbiota play a vital role in controlling food intake, nutrition, and health. However, little is known about how feed restriction, as a benefit or chronic stress, influences behavior, stress response, and gut microbial programming in slow- and fast-growing chickens. This study aimed to explore slow- and fast-growing chickens who had feed restricted to 70% of ad libitum or were given ad libitum feed for 30 days to evaluate the effects on behavior, stress response, and gut microbiota. We found that feed restriction can influence behaviors in both slow- and fast-growing breeds. Feed restriction to 70% for 30 days can influence stress response and gut microbiota composition, but some changes are evident only in slow- or only in fast-growing chickens. The study provides a better understanding of how artificial selection has affected chicken biology and their response to stress challenge.

**Abstract:**

This study aimed to explore the difference between two Chinese local broilers, one slow- and one fast-growing, in their response to a stress challenge. We conducted the study on slow- (Weining chicken) and fast-growing (Jinlinghua chicken) breeds, with 50 chickens from each breed either feed restricted to 70% for 30 days as a stress or given ad libitum to evaluate the effects on behavior, corticosterone, and microbial programming. Standing behavior was more frequent while exploration was less common in fast-growing breeds compared to slow-growing breeds. Food seeking and ingestion, exploration, and drinking increased, while resting decreased in the feed restricted treatments. There was no difference in corticosterone concentration between slow- and fast-growing chickens, but the level was affected by feeding treatments, and the interaction of breed and feed restriction. At the genus-level, the relative abundance of *Bacteroides* and *Lactobacillus* was higher, while *Cloacibacillus* and *Megasphaera* was lower in the slow-growing breed compared to the fast-growing breed. Feed restricted birds had a higher abundance of *Mucispirillum*, but lower abundance of *Cloacibacillus*, *Clostridium XlVa* and *Clostridium IV*. In conclusion, feed restriction to 70% for 30 days as a chronic stress stimulation caused more activity, elevated the stress response, and altered gut microbiota composition, but some changes were only evident in slow- or fast-growing chickens.

## 1. Introduction 

Domestication is the process by which directional selection of animals alters their phenotype to provide a function for humans. Despite thousands of years of domestication, the personality, behavioral traits and other aspects of the phenotypes of modern chickens still reflect those of their ancestral Red Junglefowl (*Gallus gallus*) [1]. China has a wide variety of indigenous domestic chickens, with 108 breeds [2] that are characterized by slow growth. The utilization of local breeds is especially popular amongst the Chinese, who think that meat/eggs from local breeds are tasty and high-quality. Notably, the local breeds are mainly used for meat production because a large number of consumers in the south of China who prefer chicken meat rather than beef and mutton as compared to north China. However, in recent decades, considerable and intentional artificial selection has accelerated the rate of domestication to develop specialty poultry for specific requirements, especially with respect to fast-growing broiler breeds (which obtain sufficient body weight for the market at approximately 5 to 7 weeks). In response to artificial selection, and changes to nutritional and other management regimes, slow- and fast-growing chicken breeds have undergone a number of changes with regard to their genetic background [3,4], growth performance [5], meat quality [6], as well as hormone secretion [7] and adaptive responses [8]. Thus, it is assumed that the stress response is also different between the slow- and fast-growing broiler breed. 

Selection for fast-growth has been sporadic, owing to concerns about welfare issues [9] such as metabolic stress and skeletal problems [10], as well as increased fat deposition due to genetic selection and large appetites [9]. Accumulating evidence indicates that feed restriction affects behavior [11] and hormone secretion [12] as well as body weight and production performance. The duration, intensity, and timing of feed restriction each have an impact on the growth of chickens [13]. For example, mild feed restriction of broilers to 90% ad libitum intake for 7 days during early life resulted in the highest body weight, while restriction to 70% for 14 days caused the lowest body weight compared to birds fed ad libitum [14]. The benefits of alternative feed restriction for chickens are controversial: some authors argue that feed restriction of broilers is commonly applied to promote animal health and decrease feed costs, and thus improve animal welfare [15]. Others argue that “metabolic hunger” exists even though restrictions promote satiety and achieve production performance [16]. Besides, the timing of appropriate feed restriction in early life is conducive to the metabolic programming and physiological process in the late stage of broilers [14,17]. The early adaptation to nutritional stress can adjust and prepare an organism for acclimation in broilers [18], although this has rarely been studied in the slow-growing local breed. It is not clear whether feed restriction to 70% for 30 days would be a benefit or a chronic stress, nevertheless, the response between the slow- and fast-growing breeds needs to be explored. 

The gut microbiota plays a vital role in the health, production performance, and welfare of chickens [19]. Researchers have gradually realized the importance of intestinal microbes to the host intestines as a whole due to their effect on many stress-related physiological functions including immunity, nutrition and metabolism [20]. The composition of the intestinal microflora can be affected by host breed, dietary interventions, housing systems, age, and stress [21,22]. A previous study indicated that laying hens undergoing feed restriction during the molting process had decreased microbial diversity, smaller *Lactobacillus* populations, and higher sensitivity to colonization by *Salmonella* [23]. In broilers, studies on the effect of feed restriction on the gut microbiota have been ambiguous [24,25]. It is probable that feed restriction, as a stressor, would alter gut microbial composition in broilers as well as local breeds. However, the effect of feed restriction as a benefit or a chronic stress that challenges the health of chickens and its impact on the gut microbiota of slow-growing versus fast-growing chicken breeds at the same age remains unknown. 

In this study, we used a native breed reared in the Wumeng Mountain Area, China, which is under relatively low selection pressure and a local Chinese breed selected for meat yield to allow a more meaningful comparison between slow- and fast-growing chickens in China. We aimed to quantify changes in body weight, behaviors, corticosterone and microbial composition in fast-growing broilers and slow-growing dual-purpose chicken genotypes at the same age under 70% feed restriction as a benefit or a chronic stress. The study provides a better understanding of how artificial selection has affected chicken biology and stress response. 

## 2. Materials and Methods 

### 2.1. Experimental Design 

The experimental protocols and animal care were approved by the China Agricultural University Laboratory Animal Welfare and Animal Experimental Ethical Inspection (approval number: CAU20180619-5). A total of 100 healthy one-day-old female Weining dual-purpose chicks (provided by Yuansheng Animal Husbandry Co., Ltd., Bijie, China; a slow-growing line with a growth rate of about 11 g/d), and 100 one-day-old female Jinling broiler chicks (provided by Nanning Jinling Agriculture and Animal Husbandry Group Co., Ltd., Nanning, China; a modern fast-growing line with a growth rate of about 27 g/d), were used. In south China, the dual-purpose local breed is mainly for meat not for laying because of the large demand. The reason for using female animals is that it excludes the gender factor. Besides, using female chickens is representative even though not comprehensive because hens are generally used for meat production rather than roosters and most farms only keep female chickens after hatching in south China, including the provinces of Guangdong, Guangxi, Hainan Island, Guizhou (where this experiment took place) and Yunnan. Our experimental animals are commercially generated by breeders in breeder farms, and the rearing standards are basically the same. The keeping and feeding of the parents were consistent, thus, this cannot influence the F1 generation. The chickens are from different farms that have mature production and rearing systems. The newly hatched birds were transported immediately to the experimental site within one hour. 

The birds were reared in a brooding barn with two enclosures (0.50 m *L* × 0.50 m *W* × 0.30 m *H*; one for each breed) where the temperature was kept above 32 °C from post-hatching until the birds were 16 days old. The 100 animals were kept in four, and six enclosures for 7 days and 21 days, respectively. Thereafter, the temperature was gradually decreased to room temperature. At the age of 27 to 29 days, each bird was moved to a single cage (0.19 m *L* × 0.30 m *W* × 0.40 m *H*) constructed on all sides with wire mesh. Birds were numbered with two 16 mm diameter foot rings on each leg and allowed to adapt to the new environmental conditions before the onset of feed restriction. Each cage had its own feeder, drinker, perch and droppings board. All chickens were randomly allocated to ad libitum or control feeding regimes to achieve a balanced sample size for each combination of breed and feeding regime. The treatments were slow-growing Weining chickens fed ad libitum (SA, n = 50) or under feed restriction (SR, n = 50), as well as fast-growing Jinling chickens fed ad libitum (FA, n = 50) or under feed restriction (FR, n = 50). Feed-restricted birds were restricted from the age of 30 days to 60 days at a level of 70% of the intake of control birds of the same breed [14]). The ad libitum feed restriction measures were estimated based on our previous study using the same breeds under cage rearing [26]. The amount of feed consumed and that left over was recorded daily for each bird. 

### 2.2. Data Collection 

#### 2.2.1. Body Weight 

The body weight of each bird was measured before feeding in the morning every week during the experimental period (31, 38, 45, 52, and 59 days of age). 

#### 2.2.2. Behavior in the Home Cage 

At 43 days of age, and after two weeks, the 70% feed restriction was deemed as a chronic stress. Chickens may adapt to the process of feed restriction treatment and the raising environment during these two weeks. In situ behaviors including standing, walking, food seeking and eating, post-feeding foraging, exploration, grooming, drinking, resting, and perching were recorded by video. To ensure the full behavioral repertoire of the birds was captured, initial scan sampling of the videos was performed to construct the ethogram in Table 1. The video recording started 1 h before feeding and lasted for 3 h from 7:00–11:00 a.m. Behaviors were thereafter estimated from scan samples taken at 10 min intervals (total of 21 scan samples per bird). Thus, the occurrence of behavior was recorded as the number of times. A single observer extracted the data from the videos. 

#### 2.2.3. Corticosterone

At 61 days, eight chickens in each treatment group were randomly selected and humanely killed. All sampled chickens were not fed before the blood collection. Blood samples were collected from 8:00~10:00 a.m. Plasma samplings were collected from 5 mL of fresh blood, immediately switched in an anticoagulation tube, centrifuged at 4000× *g* for 5 min at 4 °C and stored in 1.5 mL tube at −20 °C to prepare for the subsequent detection. The concentration of corticosterone was determined by an enzyme-linked immune sorbent assay kit (FU-J0141, China).

#### 2.2.4. Cecum Microbiota by 16S rRNA

The hatching conditions including water, feed, litter were identical, and vaccinations were given according to the growth stage of different breeds to guarantee the birds in each breed made an identical start. At 61 days of age, the cecal contents of the slaughtered birds were collected in a 1.5 mL centrifuge tube and stored at −80 °C until analysis by 16S rDNA sequencing. The cecum is by far the most densely colonized microbial habitat in chickens, and its bacterial diversity is much higher than that of the upper GI tract [22]. The most detailed information regarding chicken gut microbiota is available for the cecum, which is a key region for bacterial fermentation of non-digestible carbohydrates and the main site for colonization by pathogens [27]. 

Microbial genome DNA was extracted from cecal samples using the QIAamp DNA stool mini kit (Tiangen Biotech, Beijing, China) according to the manufacturer’s instructions. The total DNA quality was detected by a Thermo NanoDrop 2000 ultraviolet microspectrophotometer and 1% agarose gel electrophoresis. PCR amplification of the V3-V4 region of the 16S rRNA gene was performed using the 341F/806R primer set (341F: CCTACGGGRSGCAGCAG; 806R: GGACTACVVGGGTATCTAATC) as previously reported [28]. The DNA library was then sequenced by the Illumina platform (Illumina, USA). The high-quality sequences obtained were screened and uploaded to Quantitative Insights into Microbial Ecology (QIIME), v1.8.0, and clustered into operational taxonomic units (OTUs) with a sequence similarity of >97% using the USEARCH 11.0 software. 

The top 20 most abundant microbiome at the genus level were selected from the species annotation results, and the relative abundance of species was calculated according to their percentage contribution to the total microbial community (Table S1). Classification and abundance of each host treatment group was compared and analyzed. Phylogenetic Investigation of Communities by Reconstruction of Unobserved State (PICRUSt) [29] was selected to predict and calculate the functional metabolic pathways of intestinal microorganisms in different treatment groups. The raw sequencing data for the gut microbiome was deposited in the National Center for Biotechnology Information (NCBI: PRJNA664000) and released after the publication of this article. 

### 2.3. Statistical Analysis

Statistical analyses were performed with IBM SPSS Statistics 21. Body weight and corticosterone concentration met the assumptions for parametric analysis after checking for normality and homogeneity of variance and transforming where necessary. These were analyzed using a two-way ANOVA. Pairwise comparisons between treatments were performed using the LSD test option of the post-hoc test. In situ behavior observations and gut microbiota data did not meet the assumptions for parametric analysis, and therefore non-parametric methods were used. The two-way Scheirer-Ray-Hare non-parametric ANOVA analysis was used and pairwise comparisons were made using the Kruskal-Wallis test (K-W test). If the main effects were significant but interaction effects were not significant, we analyzed the main effects. If the main effects and interaction effects were both significant, we further analyzed the individual treatment level effects. Data are presented as mean ± standard deviation. All values with *p* < 0.05 are regarded as statistically significant. 

## 3. Results

### 3.1. Body Weight

**Figure 1:**
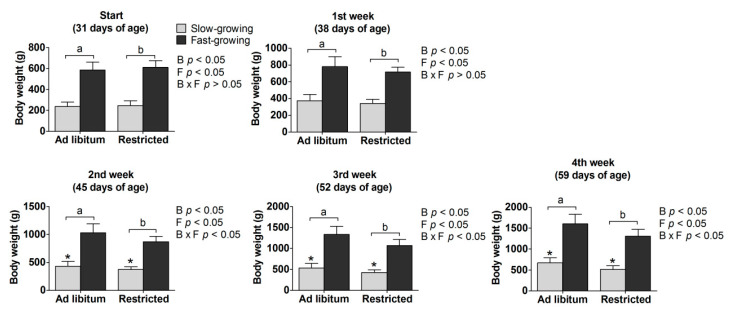
Body weight (g) of birds in the four treatments at different ages. a, b represent the significant difference between the ad libitum and feed restricted treatments (*p* < 0.05). * means significant difference in the ad libitum or feed restricted treatments between slow- and fast-growing chickens (*p* < 0.05). B: breed, F: feeding method, B × F: interaction between breed and feeding method.

Main effects: As expected, the fast-growing breed grew at a faster rate than the slow-growing breed (*p* < 0.05; Figure 1) and those fed ad libitum also grew more rapidly than those fed a restricted diet (*p* < 0.05; Figure 1). 

Interaction effect: Body weight was affected by both the breed and feeding method (ad libitum or feed restriction) (*p* < 0.05; Figure 1). 

Simple effects: Body weight was also influenced by the interaction of breed and feed restriction treatment in the time period between the second and fourth week (*p* < 0.01; Figure 1). The body weight of slow-growing feed restricted birds (SR) was lower than that of slow-growing ad libitum fed birds (SA), whereby the SR birds accounted for 90%, 86%, 79%, and 76% of the weight of the SA birds in weeks 1–4, respectively. The weight of the FR group accounted for only 92%, 84%, 80%, and 81% of that of the SA group. Here, the data was only a trend representing the weight change of the feed restriction group to ad libitum fed birds. In comparing birds on the same feeding treatment, the body weight of the FA group was higher than that of the SA group, and the body weight of the FR group was higher than that of the SR group. 

### 3.2. In Situ Behavior

**Figure 2:**
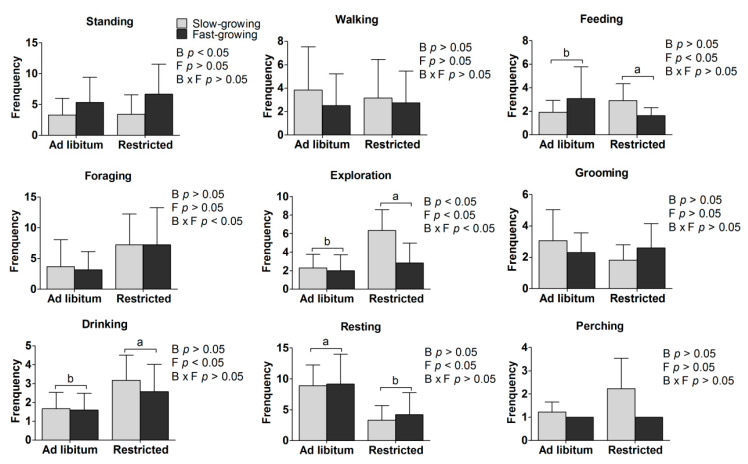
Effects of feed restriction on in situ behavior of slow- and fast-growing chickens. a, b represent the significant difference between the ad libitum and feed restricted treatments (*p* < 0.05). B: breed, F: feeding method, B × F: interaction between breed and feeding method.

In situ behavior data from the 21 scan samples (times) are shown in Figure 2.

Main effects: The incidence of standing behavior was higher and there was less exploration in fast-growing compared to slow-growing breeds (*p* < 0.05; Figure 2). The combined total of food seeking and feeding behavior, exploration, and drinking were higher, while resting was less frequent in feed restricted treatments than ad libitum treatments (*p* < 0.05; Figure 2). 

Interaction effects: Foraging and exploration were affected by both the breed and feeding method (ad libitum or feed restriction) (*p* < 0.05; Figure 2). 

Simple effects: Within the same breed, no effect of the feeding regime was found on these behaviors. No differences in these behaviors were found between the SA and the FA birds, or between the SR and FR birds. 

### 3.3. Corticosterone Concentration

**Figure 3:**
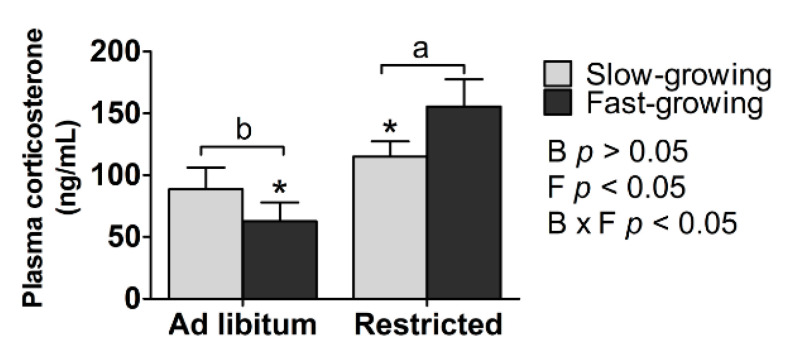
Plasma corticosterone concentration. a, b represent the significant difference between the ad libitum and feed restricted treatments (*p* < 0.05). * Means significant difference in the ad libitum or feed restricted treatments between slow- and fast-growing chickens (*p* < 0.05). B: breed, F: feeding method, B × F: interaction between breed and feeding method.

Main effects: There was no difference in plasma corticosterone concentration (ng/mL) between slow- and fast-growing chickens, but this was affected by the feeding method (*p* < 0.05; Figure 3).

Interaction effects: Plasma corticosterone concentration was affected by the interaction between breed and feeding method (*p* < 0.05; Figure 3). 

Simple effects: The corticosterone level of the SR group (1.30-fold) was significantly higher than that of the SA group, and that of the FR group (2.48-fold) was significantly higher than that of the FA group (*p* < 0.05). When comparing the two breeds given the same feeding regime, the SA group had a higher corticosterone level than the FA group, and the SR group had a lower concentration than the FR group (*p* < 0.05). 

### 3.4. Gut Microbiota

After quality control, a total of 1,080,858 clean reads were obtained, with an average of 34,866 for each sample. Read length was mainly concentrated around 380~440 bp. After cluster analysis, 785 OTUs were generated from 31 samples. On average, each sample was annotated to 407 ± 33 OTUs. 

For slow-growing chickens, 701 and 611 OTUs were obtained for the SA and SR groups, respectively, and 601 OTUs were shared in common between these treatments. For fast-growing chickens, 690 and 670 OTUs were generated in the FA and FR groups, respectively, with 625 in common. The SA and FA groups had 621 common OTUs, while SR and FR groups had 580 OTUs in common. 

#### 3.4.1. Gut Microbiome at the Genus Level 

**Figure 4:**
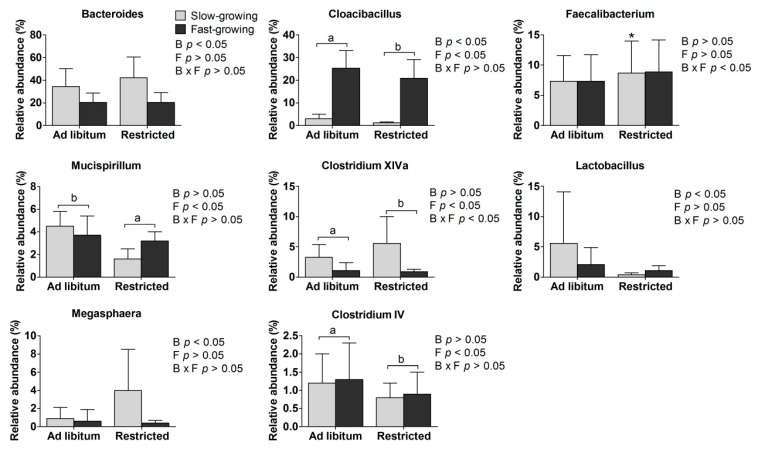
The relative abundance of the significant abundant cecal microbiomes at the genus level. a, b represent the significant difference between the ad libitum and feed restricted treatments (*p* < 0.05). * means significant difference in the ad libitum or feed restricted treatments between slow- and fast-growing chickens (*p* < 0.05). B: breed, F: feeding method, B × F: interaction between breed and feeding method.

Main effects: Compared to fast-growing chickens, the relative abundance of Bacteroides, Clostridium XlVa and Lactobacillus was higher while Cloacibacillus and Megasphaera was lower in the slow-growing breed (*p* < 0.05; Figure 4). Compared to ad libitum chickens, the relative abundance of Mucispirillum was higher, while Cloacibacillus, Clostridium XlVa and Clostridium IV was lower in the feed restricted birds (*p* < 0.05; Figure 4). 

Interaction effects: The relative abundance of Faecalibacterium and Clostridium XlVa was influenced by the breed and treatment interactions (*p* < 0.05; Figure 4). 

Simple effects: Compared to the SA group, the relative abundance of Clostridium XlVa was lower in the SR group (*p* < 0.05). The relative abundance of Faecalibacterium was lower in the FA and SR treatments as compared to the FR treatment (*p* < 0.05). 

#### 3.4.2. Functions of the Gut Microbiota

**Figure 5:**
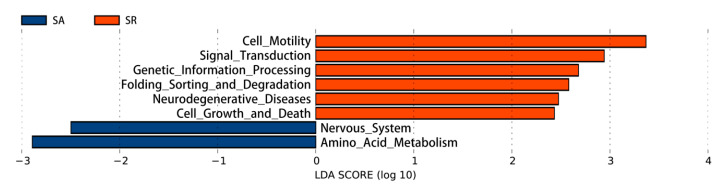
Differences in gut microbial function prediction between the SA and the SR treatments. SA: slow-growing chickens fed ad libitum, SR: slow-growing dual-purpose chickens with feed restriction.

Compared to the SA group, the SR group was enriched in pathways related to cell viability, signal transduction, genetic information processing, folding, classification and degradation, neurodegenerative diseases, and cell growth and death functions (*p* < 0.05; Figure 5). In contrast, the SA group was highly enriched in the pathways related to the nervous system and amino acid metabolism pathways (*p* < 0.05; Figure 5). However, the FA and FR treatments did not differ in the pathways that were enriched. 

**Figure 6:**
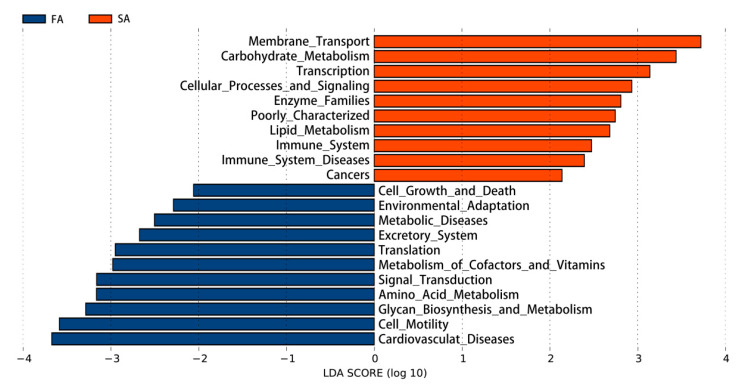
Differences in gut microbial function prediction between FA and SA treatments. SA: slow-growing dual-purpose chickens fed ad libitum, FA: fast-growing breed fed ad libitum.

Ten pathways were significantly upregulated in the SA compared to the FA group, including membrane transport, carbohydrate metabolism, transcription, cellular processes and signals, enzyme family, rare diseases, lipid metabolism, immune system, immune system diseases, and cancers. The FA group had greater enrichment in 11 pathways, including cell growth and death, environmental adaptation, metabolic disease, excretory system, translation, metabolism of coenzymes and vitamins, signal transduction, amino acid metabolism, glycine biosynthesis and metabolism, cell vitality, and cardiovascular disease (*p* < 0.05; Figure 6). 

**Figure 7:**
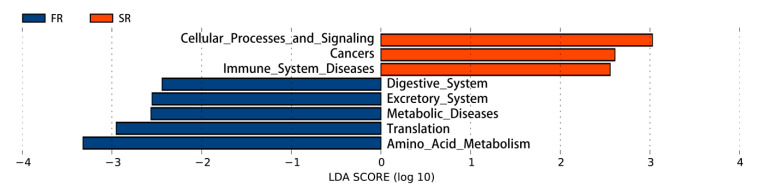
Comparison of predicted gut microbial function between the FR and SR treatments. FR: fast-growing broilers under feed restriction, SR: slow-growing dual-purpose chickens under feed restriction.

The SR group had upregulated pathways related to cellular processes and signaling, cancers, and immune system diseases as compared to the FR group. In contrast, the FR group had highly enriched functional genes in the digestive system, excretory system, metabolic diseases, translation, and amino acid metabolism pathways (*p* < 0.05; Figure 7). 

## 4. Discussion

### 4.1. Body Weight

Domestication has had profound effects on chicken productivity and artificial selection has accelerated the development of specialist poultry breeds, for example, broilers with large pectoral muscle mass and fast growth [9]. Regardless of the growth period, the fast-growing broilers gained more weight than the slow-growing chicken breed used in our study at the same age. This outcome agrees with the expected ability of fast-growing broiler breeds, such as the Ross 308 to reach 1.7 kg at 35 days, whilst slow-growing dual-purpose birds such as the Lohmann Dual are expected to reach 1.5 kg at 63 days [30]. 

The effects of feed restriction on growth depend upon its intensity, timing, and duration. Our results are consistent with those of earlier work that found that feed restriction to 70% of ad libitum intake reduces body weight [14,31]. Previous studies have shown that broilers fed at 70% of ad libitum intake suffered a 20% reduction in body weight, while those fed at 85% suffered a 12% reduction from 8 to 14 days of age compared to a control group fed ad libitum [32]. Quantitative feed restriction is regarded by some as beneficial to animal welfare because it meets one of the five freedoms of animal welfare, that is, “freedom from hunger”. However, others argue that “metabolic hunger” may still be an issue under the process of feed restriction. In our study, the growth rate appeared to be more resilient to feed restriction in the fast-growing broilers (with weights of 92%, 84%, 80%, and 81% compared to ad libitum fed birds in weeks 1–4 respectively) compared to the slow-growing chicken breed (90%, 86%, 79%, and 76%), although the differences were numerically small. The reason for the greater susceptibility of the slower-growing breed to feed restriction may be that the two breeds were at different points in their growth trajectory when the restriction was applied, with market weight being reached at 60 days of age for the broilers and 120 days for the slow-growing dual-purpose chickens. Additionally, the feed restriction duration of 30 days in our study is far beyond the duration imposed by previous studies of about 7 to 14 days. Based on the current feed restriction treatments, the weight loss in slow growers would increase. However, in our study, most of the chickens returned to normal feeding although the data were not obtained. 

### 4.2. In Situ Behaviors 

The incidence of standing behavior was higher while there was less exploration behavior in the fast-growing than the slow-growing breed. Domestication and artificial selection have changed the frequencies by which behaviors are performed rather than adding or eliminating behaviors from the behavioral repertoire. This is in contrast to the dramatic changes that have occurred in appearance, growth rate and meat quality [33,34,35]. Birds of the fast-growing breed showed less exploration behavior, which could be explained by the fast-growing broiler breed reaching maturity at 60 days of age. A previous work has shown that individuals reduced exploration with regard to the provision of heat and food after independence from the mother [36]. According to resource allocation theory, high selection pressure for production traits is likely to have resulted in a reduction in the frequency of highly energetic behaviors to allow for the investment of more energy in production performance [37]. Therefore, behavioral differences between breeds may reflect different trade-offs made to allow for the allocation of bodily resources to either growth or reproductive traits [38]. 

A significant breed and feeding treatment interaction effect indicated that exploration was around three-fold higher in the slow-growing breed under feed restriction than any of the other three treatment combinations, which suggests this aspect of behavior in the slow-growing breed is more sensitive to feed restriction. Feeding behavior was higher in the feed restricted than in the ad libitum birds, as described in a previous study [39,40]. One possibility is that ad libitum fed birds ingested less of the available food during the observation period as they were less hungry. Quantitative feed restriction can inflict feelings of chronic hunger and frustration due to unfulfilled behavioral needs for feeding [41]. Considering the higher incidence of standing behavior and lower incidence of exploration behavior in the fast-growing compared to the slow-growing breed, it seems that under feed restriction to 70% of ad libitum intake for 30 days caused more anxiety-like behavioral responses in the slow-growing breed than the fast-growing breed. Another possibility is that the feed restricted birds spent much of the one hour before feeding seeking food in the feeder, which was included in the definition of feeding used here. Foraging behaviors associated with searching for food after the available food had been consumed were higher in the restricted birds, and thus the resting behaviors were lower. 

### 4.3. Corticosterone

There was no significant effect of breed type on plasma corticosterone concentration, but this hormone did respond to feed restriction. Specifically, feed restricted birds had higher levels of corticosterone than ad libitum fed birds which is consistent with the effects of feed restriction previously reported in meat-type chickens [42] and red-legged kittiwake chicks [43]. That is, feed restriction produced higher chronic stress-induced concentrations of corticosterone compared to the ad libitum fed chicks. Corticosterone is one of the indicators used to assess stress and welfare [15]. Quantitative feed restriction may inflict chronic hunger, frustration and stress due to inadequate feeding [41]. The level of corticosterone can be regarded as one of the physiological stress parameters in feed restriction [44,45,46,47]. Thus, the corticosterone concentration is collected to assess the stress-related response to feed restriction [48]. Quantitative feed restriction may cause a normal physiological response or result in physiological or psychological stress. Generally, quantitative feed restriction results in signs of stress in terms of high concentrations of plasma corticosterone in broilers [41,42,47,49,50]. A previous study indicates that corticosterone plays a direct role in promoting food-searching behavior under conditions of nutritional stress through feed restriction treatments [51], which may result in a higher corticosterone concentration and increased feeding behavior in feed restricted group than ad libitum group. 

In our study, the corticosterone concentration in the SA treatment was higher than that in the FA treatment, yet that in the FR group was higher than in the SR group. The level of corticosterone in the FR group was 2.48-fold higher than that in the FA group, whilst the concentration in the SR group was 1.30-fold higher than in the SA group. These fold changes would suggest that corticosterone secretion is particularly sensitive to feed availability in the fast-growing breed. The reason for this breed difference is unclear, but breed differences in response to other forms of stress have previously been reported (e.g., in red junglefowl and domestic White Leghorns [52]). Our results indicate that despite the fast-growing birds being able to maintain their growth rate more successfully than the slow-growing birds when feed restricted, they may experience metabolic stress, which has a potentially negative impact on welfare [8]. Therefore, feed restriction to 70% as a diet intervention may cause nutritional stress in these birds. 

### 4.4. Gut Microbiota

There was a difference in the gut microbiota of slow- and fast-growing breeds, which is in line with previous reports of breed effects [21,53]. Considering the main effect of breed, the relative abundance of *Bacteroides* and *Lactobacillus* was higher in the slow-growing breed. It has been reported that body weight gain is affected by gut microbial composition [54]. *Bacteroides* are found in lower abundance in fat line chickens compared to lean line birds [55], which is consistent with fast-growing broilers having a lower abundance of *Bacteroides*. Conversely, the abundance of *Cloacibacillus* was higher in the fast-growing breed than the slow-growing breed, which is conducive to a fast growth rate [56]. *Lactobacillus*, as one of the health-promoting microbiomes [57], had higher relative abundance in the slow-growing breed than in the fast-growing breed. 

Feed restriction was associated with higher *Mucispirillum* presence accompanied by lower body weight, which agrees with an earlier work linking the increased population of *Mucispirillum schaedleri* with lower body weight [58]. In contrast, the relative abundance of *Cloacibacillus*, *Clostridium XlVa*, and *Clostridium IV* was lower in the feed restricted than ad libitum fed birds. The *Clostridium XlVa* and *IV* clusters of the *Firmicutes* phylum are major components of the chicken cecal microbiota and play a positive role in growth performance [27,59]. Our results conflict with those of earlier studies in some regards, perhaps due to the different duration and severity of feed restriction imposed. For example, previous work found that feed restriction decreased *Lactobacillius* populations in the gut of laying hens [23], which was not found in our work. Also, a quantitative feed restriction of 87.5% and 75% of ad libitum intake imposed for 7 and 14 days, respectively [23], or of 50% for 7 days [24] was found to have a limited effect on the cecum microbiota in broilers, whilst our study did find such effects. Thus, the gut microbiota appears to be sensitive to longer duration feed restriction and an intensity of 70% of ad libitum intake for 30 days is sufficient to induce these changes. 

The relative abundance of *Faecalibacterium* and *Clostridium XlVa* was influenced by the interaction of breed and feeding treatment. *Faecalibacterium*, a putatively beneficial genus belonging to the *Firmicutes* phylum, had higher relative abundance in the FA and SR groups than the FR group. This genus is associated with butyrate production to enhance the capacity to harvest energy [59], and with the obesity phenotype in humans and mice [60]. *Faecalibacterium* was seemingly not associated with the obesity phenotype but rather with tolerance to feed restriction, suggesting that slow-growing birds were more tolerant of restriction than fast-growing birds, which mirrors the greater sensitivity of corticosterone levels to feed restriction in the FR group. The predominant *Clostridium XlVa* cluster had higher abundance in the SA than the SR group. This is a significant butyrate-producing cluster that contributes to gut growth and microbial composition balance in the chicken cecum [59]. 

The gut microbiota performs a large number of roles for the host through functional microbial pathways. Feed restriction modulated the effect of the microbiota on the neural system as indicated by differential effects on the nervous system and neurodegenerative diseases in the SA and SR groups. Feed consumption is closely associated with the regulation of the central nervous system [61]. Here, it seems that feed restriction to 70% of ad libitum intake for 30 days is not conducive to the neural-related development in the slow-growing breed. Whilst no differences in pathway enrichment were found between fast-growing birds given an ad libitum or restricted diet, eight differently enriched pathways were found between slow-growing breeds on the two diets. The discrepancy between breeds may be due to them being at different points of their growth trajectory as described above with respect to effects on body weight. Specifically, slow-growing birds were still well within the growth development phase and the microbial functions may thus have been more easily affected by the feed restriction, while fast-growing birds were at a marketable age and therefore more mature and potentially with more stable microbial functions. More work would be needed to confirm this suggestion. Furthermore, the gut microbiota is associated with the neural system through its critical role in the gut-brain axis [62]. It was noted that 10 pathways were up-regulated and 11 were down-regulated in the SA group compared to the FA group. The immune system and immune system disease pathways were upregulated in the SA group compared to FA group. Up-regulated cancer and immune-related disease pathways found in the slow-growing dual-purpose birds may be due to them being at an early developmental stage with a fragile immune system compared to the rapidly maturing broilers [63]. Additionally, we found enrichment of the cardiovascular disease pathway in the FA group compared to the SA group. As documented, fast-growing broilers are highly susceptible to cardiovascular disease [64], cardiac arrhythmia [65], and sudden death syndrome [64], as well as skeletal problems [10]. Importantly, the incidence of cardiovascular disease can be reduced when the growth rate of the fast-growing broilers is manipulated and controlled by feed restriction [65]. Besides, metabolic disease is more frequent in fast-growing breeds compared to slow-fasting breeds. This implies that the process of artificial selection causes welfare problems [9] and metabolic stress [10]. As a result of feed restriction, the immune system disease pathway was enriched in the SR group and the metabolic disease pathway was enriched in the FR group, which should be considered when adjusting the timing, intensity and duration of feed restriction for slow- and fast-growing chickens. In addition, the digestive system pathway was enriched in the FR group compared to the SR group. The gut microbiota plays a vital role in the process of nutrient digestion and absorption. That is, microbial communities in broilers were better able to consume, store, and circulate nutrients effectively under feed restriction [66]. 

## 5. Conclusions

Feed restriction decreased the body weight in both the slow- and fast-growing breeds and affected some aspects of behavior, but mostly not in a breed-specific way. The fast-growing breed had a lower stress tolerance than the slow-growing breed during the feed restriction as indicated by elevated corticosterone concentrations. As a chronic stress, feed restriction to 70% of ad libitum intake for 30 days influenced the composition of the gut microbiota in chickens, and the functional pathways that were affected indicated that the fast-growing breed benefited from the feed restriction. Intentional selection has caused different phenotypic responses to the feed restriction regime imposed here in slow- and fast-growing breeds with respect to body weight, stress response, and gut microbiota. 

## Figures and Tables

**Table 1:** Classification and definition of in situ behavior.

Behavior Classification	Behavior Definition
Standing	Standing with legs upright without locomotion.
Walking	Locomotion at a normal speed.
Exploration	Pecking around the cage wall, the bottom of the cage or scratching the ground before feeding; looking around in a non-stationary state.
Food seeking and feeding	Searching for food from the feeder before feed was provided and ingestion of food after its provision.
Foraging	Pecking around the cage wall, the bottom of the cage or the area around the feeder after feeding but without ingesting food.
Grooming	Preening feathers with beak or claws, combing the feathers, or stretching the wings and legs.
Drinking	Tapping the nipple drinking fountain.
Resting	Lying down whilst performing none of the other defined behaviors.
Perching	Standing or squatting motionless on the perch.

## Data Availability

The raw data of gut microbiota presented in this study are openly available in the National Center for Biotechnology Information (NCBI: PRJNA664000).

## Supplementary Materials

The following are available online at https://www.mdpi.com/2076-2615/11/1/141/s1, Table S1: The relative abundance of the 20 most abundant cecal microbiomes among four groups $\bar{X}\pm SE$.
