# Supplementary material for: Feed Restriction Induced Changes in Behavior, Corticosterone, and Microbial Programming in Slow- and Fast-Growing Chicken Breeds
Source: Animals (Basel). 2021 Jan 11;11(1):141. doi: 10.3390/ani11010141 (PMC7827424; doi:10.3390/ani11010141)
Supplement: Supplementary file 1 [file animals-11-00141-s001.pdf]

animals

MDPI

Table S1. The relative abundance of the 20 most abundant cecal microbiomes among four groups ($\bar{X} \pm SE$).

Simple Effects	Breed Level Effects				Feed Method Level Effects				K-W Test
Genus	SA	FA	SR	FR	SA	SR	FA	FR	p-value
Bacteroides	14.8 ± 2.7	6.6 ± 1.2	22.5 ± 3.7	7.3 ± 1.2	14.8 ± 2.7	22.5 ± 3.7	6.6 ± 1.2	7.3 ± 1.2	-
Cloacibacillus	1.1 ± 0.2	7.4 ± 0.6	0.6 ± 0.1	7.6 ± 1.3	1.1 ± 0.2	0.6 ± 0.1	7.4 ± 0.6	7.6 ± 1.3	-
Faecalibacterium	3.6 ± 0.6	1.6 ± 0.4	2.4 ± 0.5 [b]	4.2 ± 0.5 [a]	3.6 ± 0.6	2.4 ± 0.5	1.6 ± 0.4 [B]	4.2 ± 0.5 [A]	0.005
Megamonas	1.9 ± 0.6	1.0 ± 0.3	6.1 ± 3.4	2.1 ± 0.7	1.9 ± 0.6	6.1 ± 3.4	1.0 ± 0.3	2.1 ± 0.7	-
Prevotella	2.5 ± 0.9	1.4 ± 0.2	1.0 ± 0.2	2.3 ± 0.6	2.5 ± 0.9	1.0 ± 0.2	1.4 ± 0.2	2.3 ± 0.6	-
Desulfovibrio	1.6 ± 0.3	1.6 ± 0.4	2.3 ± 0.3	2.0 ± 0.3	1.6 ± 0.3	2.3 ± 0.3	1.6 ± 0.4	2.0 ± 0.3	-
Alistipes	1.6 ± 0.3	1.6 ± 0.5	1.9 ± 0.6	1.7 ± 0.2	1.6 ± 0.3	1.9 ± 0.6	1.6 ± 0.5	1.7 ± 0.2	-
Mucispirillum	0.8 ± 0.3	0.9 ± 0.5	3.1 ± 0.8	0.7 ± 0.2	0.8 ± 0.3	3.1 ± 0.8	0.9 ± 0.5	0.7 ± 0.2	-
Clostridium XlVa	1.8 ± 0.1	1.1 ± 0.1	0.9 ± 0.2	1.1 ± 0.1	1.8 ± 0.1 [A]	0.9 ± 0.2 [B]	1.1 ± 0.1	1.1 ± 0.1	0.006
Lactobacillus	1.4 ± 0.3	0.3 ± 0.1	3.0 ± 1.0	0.3 ± 0.1	1.4 ± 0.3	3.0 ± 1.0	0.3 ± 0.1	0.3 ± 0.1	-
Olsenella	2.6 ± 1.5	0.6 ± 0.2	0.2 ± 0.1	0.4 ± 0.1	2.6 ± 1.5	0.2 ± 0.1	0.6 ± 0.2	0.4 ± 0.1	-
Barnesiella	1.0 ± 0.6	0.6 ± 0.3	2.0 ± 1.3	0.2 ± 0.0	1.0 ± 0.6	2.0 ± 1.3	0.6 ± 0.3	0.2 ± 0.0	-
Megasphaera	0.5 ± 0.2	1.1 ± 0.3	0.6 ± 0.3	0.6 ± 0.1	0.5 ± 0.2	0.6 ± 0.3	1.1 ± 0.3	0.6 ± 0.1	-
Campylobacter	0.3 ± 0.2	0.3 ± 0.2	2.2 ± 1.0	0.1 ± 0.0	0.3 ± 0.2	2.2 ± 1.0	0.3 ± 0.2	0.1 ± 0.0	-
Methanobrevibacter	1.3 ± 0.7	0.4 ± 0.2	0.2 ± 0.1	0.2 ± 0.1	1.3 ± 0.7	0.2 ± 0.1	0.4 ± 0.2	0.2 ± 0.1	-
Clostridium IV	0.7 ± 0.1	0.5 ± 0.0	0.3 ± 0.0	0.4 ± 0.0	0.7 ± 0.1	0.3 ± 0.0	0.5 ± 0.0	0.4 ± 0.0	-
Butyricicoccus	0.5 ± 0.1	0.5 ± 0.2	0.4 ± 0.1	0.3 ± 0.1	0.5 ± 0.1	0.4 ± 0.1	0.5 ± 0.2	0.3 ± 0.1	-
Paraprevotella	0.3 ± 0.1	0.4 ± 0.1	0.3 ± 0.2	0.2 ± 0.1	0.3 ± 0.1	0.3 ± 0.2	0.4 ± 0.1	0.2 ± 0.1	-
Oscillibacter	0.3 ± 0.0	0.2 ± 0.0	0.3 ± 0.1	0.2 ± 0.0	0.3 ± 0.0	0.3 ± 0.1	0.2 ± 0.0	0.2 ± 0.0	-
Helicobacter	0.1 ± 0.0	0.3 ± 0.1	0.4 ± 0.2	0.3 ± 0.1	0.1 ± 0.0	0.4 ± 0.2	0.3 ± 0.1	0.3 ± 0.1	-
Other	61.4 ± 3.2	71.7 ± 2.1	49.4 ± 1.8	67.7 ± 2.3	61.4 ± 3.2	49.4 ± 1.8	71.7 ± 2.1	67.7 ± 2.3	-

Note: [a, b] represents the significant difference between slow- and fast-growing breeds in the same treatment. [A, B] represents the significant difference between ad libitum vs feed restriction in the same breed. SA: slow-growing dual-purpose chickens fed ad libitum, SR: slow-growing dual-purpose chickens with feed restriction, FA: fast-growing broilers fed ad libitum, FR: fast-growing broilers with feed restriction.
